# Supplementary figures and images for: Representation learning of genomic sequence motifs with convolutional neural networks
Source: PLoS Comput Biol. 2019 Dec 19;15(12):e1007560. doi: 10.1371/journal.pcbi.1007560 (PMC6941814; doi:10.1371/journal.pcbi.1007560)

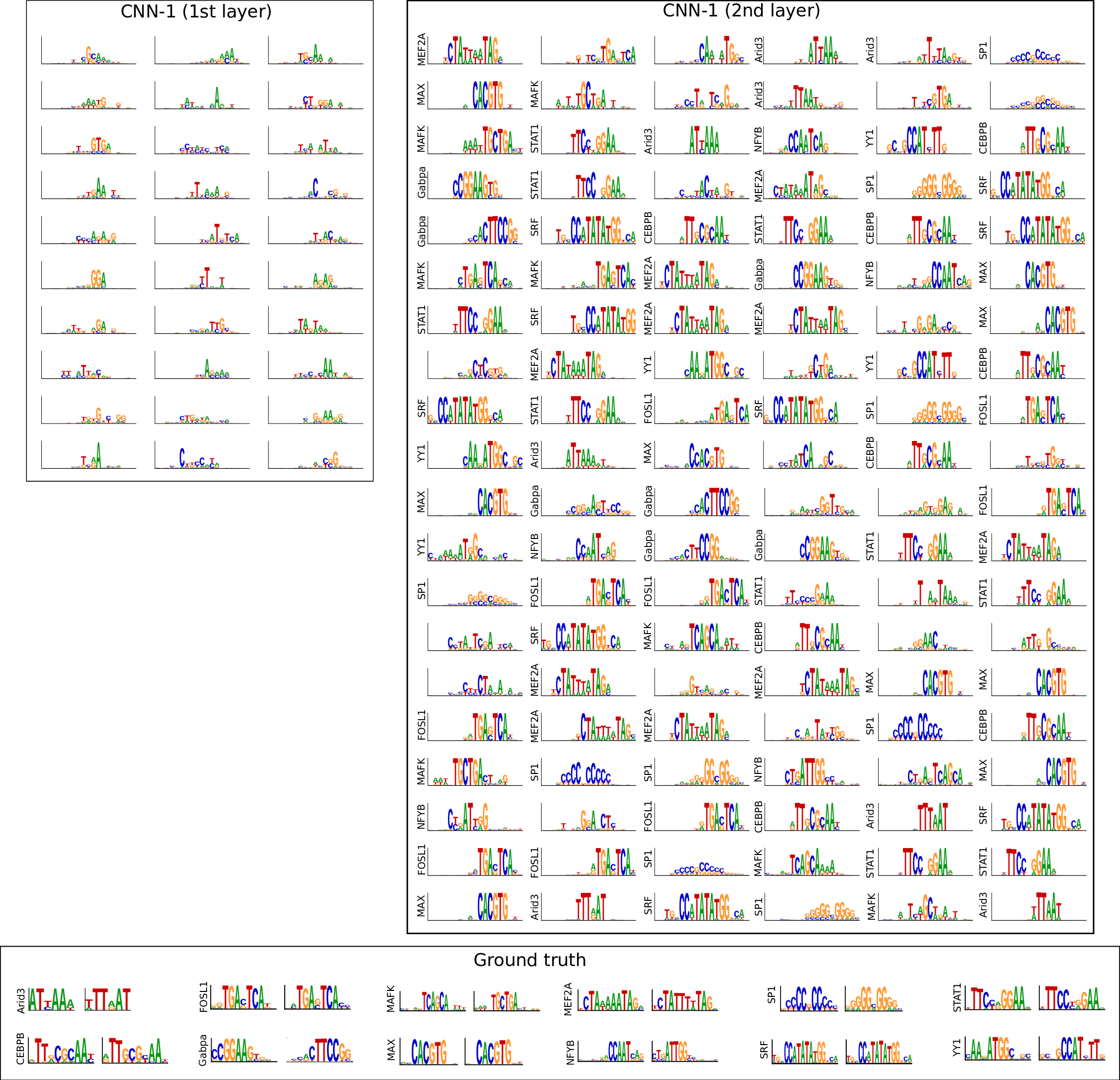

Supplement: S1 Fig — Sequence logos for first convolutional layer filters (left) and second convolutional layer filters (right) are shown for CNN-1 trained on synthetic sequences. The sequence logos of ground truth motifs and their reverse complements for each transcription factor from the JASPAR database is shown at the bottom. The y-axis label on select filters represent a statistically significant match to a ground truth motif. (TIF) [file pcbi.1007560.s001.tif]

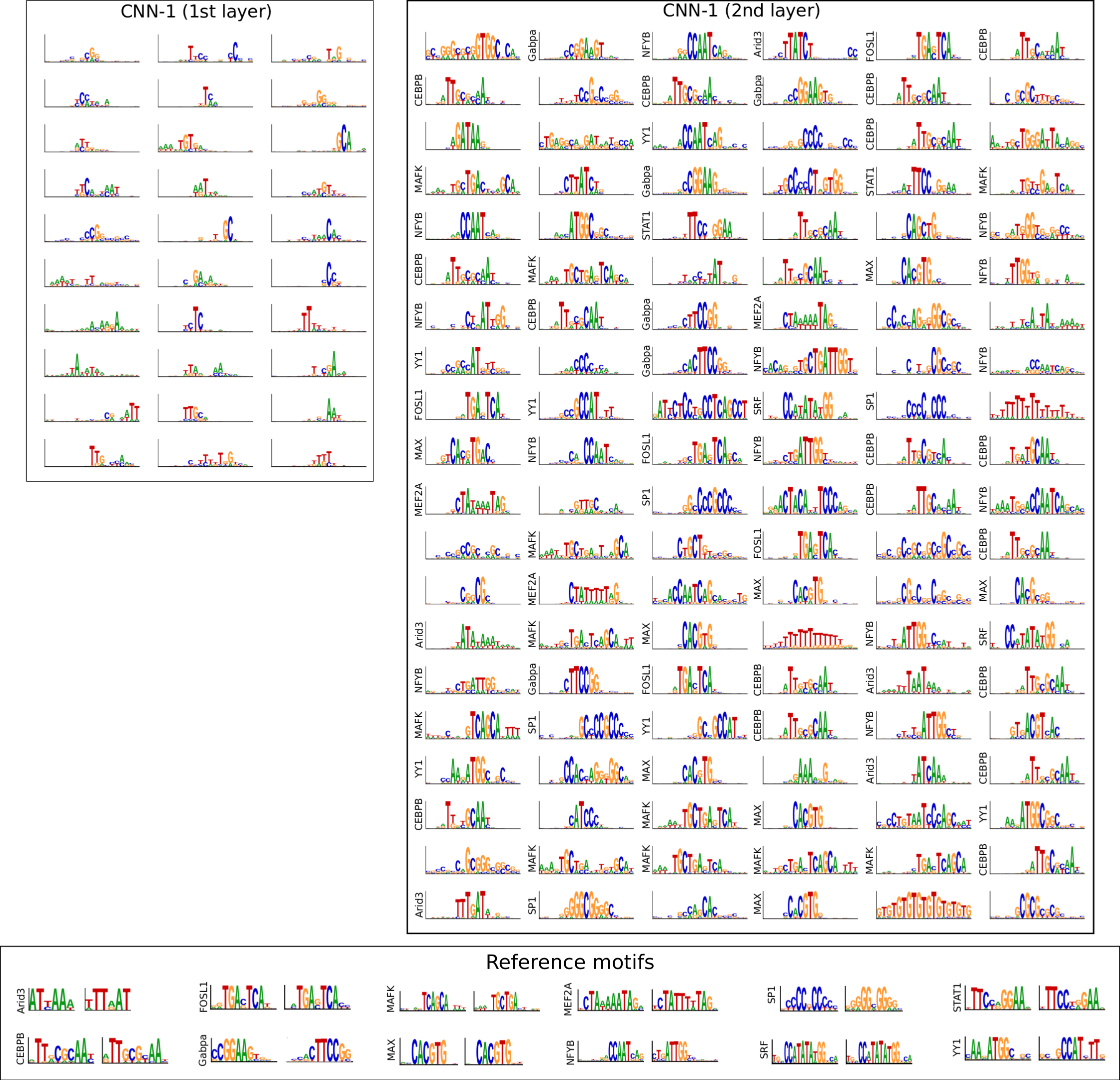

Supplement: S2 Fig — Sequence logos for first convolutional layer filters (left) and second convolutional layer filters (right) are shown for CNN-1 trained on in vivo sequences. The sequence logos of reference motifs and their reverse complements for each transcription factor from the JASPAR database is shown at the bottom. The y-axis label on select filters represent a statistically significant match to a reference motif. (TIF) [file pcbi.1007560.s002.tif]
