# Supplementary material for: Representation learning of genomic sequence motifs with convolutional neural networks
Source: PLoS Comput Biol. 2019 Dec 19;15(12):e1007560. doi: 10.1371/journal.pcbi.1007560 (PMC6941814; doi:10.1371/journal.pcbi.1007560)
Supplement: S1 Table — The table shows the class index from the DeepSea dataset (zero-based index), the transcription factor name, the cell type, the number of training sequences, and the original ENCODE file name for each transcription factor in the in vivo dataset. (PDF) [file pcbi.1007560.s003.pdf]

Table S1: Details of the *in vivo* dataset. The table shows the class index from the DeepSea dataset (zero-based index), the transcription factor name, the cell type, the number of training sequences, and the original ENCODE file name for each transcription factor in the *in vivo* dataset.

Index	Name	Cell type	# of sequences	ENCODE file name
592	ARID3A	K562	27652	wgEncodeAwgTfbsSydhK562Arid3asc8821IggrabUniPk.narrowPeak.gz
602	CEBPB	K562	85354	wgEncodeAwgTfbsSydhK562CebpbIggrabUniPk.narrowPeak.gz
344	FOSL1	K562	19724	wgEncodeAwgTfbsHaibK562Fosl1sc183V0416101UniPk.narrowPeak.gz
345	GABP	K562	34194	wgEncodeAwgTfbsHaibK562GabpV0416101UniPk.narrowPeak.gz
635	MAFK	K562	43528	wgEncodeAwgTfbsSydhK562Mafkab50322IggrabUniPk.narrowPeak.gz
636	MAX	K562	87290	wgEncodeAwgTfbsSydhK562MaxIggrabUniPk.narrowPeak.gz
349	MEF2A	K562	9792	wgEncodeAwgTfbsHaibK562Mef2aV0416101UniPk.narrowPeak.gz
642	NFYB	K562	22758	wgEncodeAwgTfbsSydhK562NfybUniPk.narrowPeak.gz
359	SP1	K562	17450	wgEncodeAwgTfbsHaibK562Sp1Pcr1xUniPk.narrowPeak.gz
361	SRF	K562	7528	wgEncodeAwgTfbsHaibK562SrfV0416101UniPk.narrowPeak.gz
661	STAT1	K562	4516	wgEncodeAwgTfbsSydhK562Stat1Ifng30UniPk.narrowPeak.gz
369	YY1	K562	31146	wgEncodeAwgTfbsHaibK562Yy1V0416101UniPk.narrowPeak.gz
